# Psychometric evaluation of the German version of the Patient Satisfaction with Cancer-related Care questionnaire

**DOI:** 10.1186/s12913-020-05838-7

**Published:** 2020-10-27

**Authors:** Frederike Bokemeyer, Lukas Lange-Drenth, Pascal Jean-Pierre, Holger Schulz, Christiane Bleich

**Affiliations:** 1 Department of Medical Psychology, University Cancer Center Hamburg, University Medical Center Hamburg-Eppendorf, Martinistraße 52, Hamburg, Hamburg Germany; 2 Florida State University College of Medicine, Cancer Neurocognitive Translation Research Lab, Tallahassee, Florida USA

**Keywords:** Patient satisfaction, Cancer, Psychometrics, Cancer care, Quality improvement

## Abstract

**Background:**

Patient satisfaction is a fundamental aspect of perceived health care quality. The original English version of the Patient Satisfaction with Cancer-related Care (PSCC) is a psychometrically validated, one-dimensional instrument with relevance to cancer-related care. The goal of the study was to perform a psychometric validation of the PSCC in German (PSCC-G).

**Methods:**

A sample of 394 cancer patients were recruited at oncological clinics in Hamburg, Germany. Patients completed the PSCC-G, three subscales of the Patient Satisfaction and Quality in Oncological Care (PASQOC), and one subscale from the German version of the Recherché Evaluative sur la Performance des Réseaux de Santé (RESPERE-60) questionnaire. We conducted exploratory and confirmatory factor analyses (EFA and CFA) to determine the factorial validity, and we calculated Cronbach’s coefficient alpha (*α*) to test the internal consistency of the PSCC-G. We examined the correlation between the PSCC-G and four subscales measuring additional dimensions of PS with care. We also conducted a multiple linear regression analysis to determine whether sociodemographics, self-perceived health status, and treatment setting predict scores on the PSCC-G.

**Results:**

The EFA (using principal axis) revealed a one-factor solution. The *Cronbach’s α* was 0.92. The convergent validity showed high correlations between three different subscales measuring patient satisfaction and the PSCC-G. Overall, males, older age patients, and those with a higher self-perceived health status were more satisfied with their cancer care based on their higher scores on the PSCC-G.

**Conclusion:**

The PSCC-G is a reliable and valid instrument that can assess satisfaction with cancer-related care for German-speaking cancer patients.

**Supplementary Information:**

The online version contains supplementary material available at 10.1186/s12913-020-05838-7.

## Background

Patients’ perception is crucial for assessing the quality of health care and essential for providing insight into the impact of diagnosis and therapy on the patient. Patient-reported measures are of importance to the evaluation of the quality of health care provided. Patients’ perception of the quality of health care they received is generally determined based on two broad dimensions: patient experience and patient satisfaction (PS) [1–3]. Satisfaction measures can provide insights that will facilitate the integration of patients’ viewpoints into their treatment processes. Longitudinal assessment of PS can help policymakers make more relevant decisions for resource allocation, inform the evaluation of the effectiveness of health care programs. Additionally, PS instruments can serve as tools for benchmarking the quality of care in clinics and medical practices [2, 4].

PS is an outcome measure of a patient’s experiences of care that reflects whether or not the care provided has met the patient’s needs and expectations [2, 5]. Previous studies have identified various associated factors that can influence PS with cancer care including age [6], education [7], marital status [8, 9] and quality of life [10] as well as medical parameters such as self-rated health status [7, 11], and types of treatment [11]. Other studies have reported significant associations between PS and cancer treatment outcomes [7, 12–14]. Specifically, patients who are satisfied with their cancer care are more likely to adhere to treatment and care follow-up recommendations [15], actively engage in the treatment process and participate in decisions concerning their cancer care [16].

A variety of different questionnaires measure PS with cancer care. These questionnaires generally focused on a particular type of cancer [17], a specific cancer stage [18, 19], a single treatment type, or one treatment environment [20, 21]. None of these questionnaires spans the spectrum of cancer-related care from screening to treatment. However, the Patients Satisfaction with Cancer Care (PSCC) and the Spanish version (PSCC-SP) are brief, psychometrically validated, one-dimensional instruments with relevance to patients receiving diagnostic and therapeutic cancer-related care regardless of their type of cancer [22, 23]. The goal of this study was to complete the linguistic and psychometric validation of the PSCC for German-speaking patients receiving cancer-related care for different types of malignancies at inpatient or outpatient cancer clinics.

## Methods

### Study design

This study is a psychometric validation analysis of the German version of the PSCC. The Ethics Committee of the Medical Association in Hamburg, Germany (tracking number: PV5785) approved this study. We implemented this study per the Code of Ethics of the Declaration of Helsinki.

### Translation and cultural adaptation of the PSCC for German speakers

We followed a standardized procedure described by the European Organization for Research and Treatment of Cancer (EORTC) quality of life group to translating the original English version of the PSCC [22] into German (PSCC-G) [24]. Three of the authors (native German speakers) independently translated the items from English to German. The fourth translator reconciled all three versions of the questionnaire into one final version. Two native English speakers independently back-translated the reconciled version into English. The two English back-translated versions were compared to the original version by three of the authors, and any deviations were discussed and resolved among all authors.

### Pilot testing

We pilot-tested the PSCC-G with the complete set of validation questionnaires in April 2018 in five cancer patients we recruited from the oncology ward of the University Medical Center Hamburg-Eppendorf (UKE). The inclusion criteria for participation in the pilot test were identical to those of the main study. Participants were instructed to think aloud while completing the questionnaires to identify how items are interpreted, whether instructions are easy to understand, whether problems occur and whether participants can understand the items in the way the researcher intended [25]. The pilot study revealed that patients generally understood the PSCC-G well.

### Procedure and participants

We recruited participants from the oncological wards and outpatient clinics (II. Medical Clinic and Polyclinic, Department of Radiotherapy and Radiation Oncology, the Department of Gynecology and the breast cancer center) of the University Cancer Center Hamburg (UCCH) as well as at both locations of the hematological-oncological outpatient clinic Altona, Hamburg, from May to June 2018. Patients in outpatient treatment were addressed by a researcher while waiting for their appointment. Those in inpatient treatment were approached by a researcher in their room on the ward. In both settings, the researcher consulted the clinical staff first to get information on who fulfilled the inclusion criteria and was in a physical and mental state to participate in the study. During the completion of the questionnaire, participants were able to ask questions, submit complaints, or give feedback. Patients were encouraged to contact the researcher if they have any remaining questions after completing the questionnaire using the contact information included in the informed consent forms. All participants read and provided signed informed consent before participating in the study. The inclusion criteria for this study were: age > 18 years; diagnosis of cancer, and sufficient written and spoken knowledge of the German language to understand the study questionnaires.

### PSCC-G

Similarly to the original English version of the PSCC, the German version (i.e., PSCC-G) consists of 18 items. Participants responded to the PSCC on a 5-point Likert scale that ranged from ‘1 = strongly agree’ to ‘5 = strongly disagree’ [22]. We inverted the direction of the response scale to range from ‘1 = strongly disagree’ to ‘5 = strongly agree’ for the PSCC-G based on participants’ feedback from our pilot study. The original English PSCC is a one-dimensional scale and explained 62% of the variance in satisfaction with cancer-related care [22]. The original versions of the PSCC in English and Spanish (i.e., PSCC-SP) are both reliable and valid measures [22, 23].

### Additional measures

We used subscales from the following validated self-report questionnaires to measure additional dimensions of cancer patients’ satisfaction with care. The subscale ‘satisfaction with information provided by physicians of the Recherché Evaluative sur la Performance de Réseau de Santé-German version (RESPERES-G) questionnaire was used to measure informational satisfaction. The RESPERES-G is a German translation of a French questionnaire [26] that was psychometrically validated with German cancer patients (Sautier et al., submitted). The subscale ‘satisfaction with information provided by physicians’ had a good internal consistency (α = .88). All items were scored on a five-point-response scale ranging from ‘1 = bad’ to ‘5 = excellent’.

We used the following three subscales from the German version of the Patient Satisfaction and Quality in Oncological Care (PASQOC) questionnaire [9, 27]: 1) co-management and shared decision making; 2) nursing staff and other practice assistants; 3) involvement of family members and friends. The majority of items referred to the experience of the patient. The answers were on different nominal or ordinal scales (e.g., If you had questions, did you get answers you could understand? The response options were Yes, always – Sometimes – No – I did not need/want this). Additionally, some of the items were rating questions (e.g., How would you rate the compassion and kindness of the nurse towards you? The response options included Bad – Reasonable – Good - Very good – Excellent) [9, 27]. We changed the response options of the three subscales to a five-point response scaling that ranged from ‘1 = I strongly agree’ to ‘5 = I strongly disagree’ as recommended by participants from our pilot study.

Additionally, we assessed the sociodemographic and clinical characteristics of participants. Sociodemographic variables included age, sex, marital status, education, and professional situation. The clinical characteristics variables included the types of cancer, treatment modalities, comorbidity, and self-perceived health status) of the participants. Participants’ self-perceived health status was determined using the global assessment health (“How would you rate your overall health during the past week”) of the German Version of the EORTC QLQ-C30 [28]. Appendix 1 contains an overview of all of the instruments and subscales used.

### Data analysis

We used IBM SPSS Statistics version 25 for our statistical analyses. We excluded cases with more than 30% of the PSCC-G items missing, leading to the exclusion of 19 cases [29]. We used descriptive statistics (i.e., means, standard deviations (SD), frequencies) to characterize the sample. We assessed item properties, internal consistency, factorial validity, and convergent validity to determine the psychometric properties of the PSCC-G, assessed. Item analysis included item means, SD, acceptance (% missing per item) [30], skewness (+ 3, − 3 thresholds [31]), the observation of a ceiling effect (i.e., cut-off per item = 50% or more answering with the maximum value), and the corrected item-total correlations (.40 threshold) [32]. We imputed missing values using the expectation-maximization algorithm.

We calculated Cronbach’s coefficient alpha to determine the internal consistency of the PSCC-G.

For the evaluation of the factorial structure we primarily performed a confirmatory (CFA) and – in case of an insufficient model fit – a complementary exploratory factor analysis (EFA). The model for the CFA was based on the factorial structure of the PSCC and PSCC-Sp [22, 23]. To assess the goodness of fit of the data to the original one-dimensional model, we performed a maximum likelihood CFA. We examined the model chi-square, the comparative-fit-index (CFI), the root mean square error of approximation (RMSEA), the Standardized Root Mean Square Residual (SRMR) and the Tucker–Lewis Index (TLI). The CFI and TLI should be close to or > .95, the SRMR is recommended to be close to or < .09 and the RMSEA should be < .06 [33]. We also examined the factor loadings, following Comrey and Lee’s [34] recommendations (i.e.*,* ≥ .71 = excellent, .64–.70 = very good, .55–.63 = good, .46–.54 = fair, and .32–.45 = poor). Factor loadings below .32 were regarded as too low because such factor can only explain approximately 10% of the variance in the variable. Hence, we would not have considered these items further [34]. The CFA was performed using IBM-AMOS 24.0. Since the predicted one-factor structure of the original English version [22] was not fully confirmed by the CFA, an additional EFA was conducted. Principal axis analysis was selected as the extraction method for the EFA. The number of factors to be extracted was determined by factor eigenvalues (λ) above 1.0 and examination of the scree-plot. Factor loadings for the EFA were also interpreted using Comrey and Lee’s [34] recommendations. We used the Kaiser–Meyer–Olkin value (KMO) in determining the suitability of the data [35].

To measure convergent validity, we calculated correlations (Pearson’s correlation coefficient) between the PSCC-G and the subscales satisfaction with information provided by physicians of the RESPERES-G questionnaire and the PASQOC subscales co-management, shared decision making, nursing staff, and other practice assistants, and involvement of family members and friends.

In the multiple regression analysis we investigated whether socio-demographic (age, sex, education, marital status) or medical variables (self-perceived health status and treatment setting), predict PS with cancer-related care (PSCC-G total score). The categorical variables were dummy coded. For categorical variables with more than two characteristics, we chose one base-category that was not included in the model. We conducted a priori power calculation for the planned multiple regression analyses by using G*Power [36]. Based on this analysis, we concluded that data from 400 patients must be available for the study. This number of patients was sufficient to demonstrate a correlation with small to a medium effect size of R = .20 with an 80% power and a level of significance set at alpha = 0.05 in a multiple linear regression analysis with 10 predictor variables.

## Results

### Results of the translation

For the most part, the two English back translations of the reconciled German version were similar to the English original. Only small adjustments seemed to be necessary for items 11, 12, and 14. These three items were simplified to more appropriately match lower literacy levels. The translation of generic terms with multiple meanings and no exact equivalent in the German language, such as ‘care’ (items 9, 10, 14, 15, 16, and 17) were the most challenging. The terms used in German translation were treatment (Behandlung) and services (Leistungen).

### Sociodemographic and medical characteristics of the sample

Overall, 394 participants with a mean age of 57.1 years (range 19–85) participated in the study (Table 1). Slightly more males (57%) than females (43%) participated in the study. About one-third of participants had a University degree (32%). Most of the participants were married (64%) or lived in a committed relationship. The most often reported cancer diagnosis in this sample were lymphoma (15%), breast cancer (13%), and prostate cancer (13%). The majority of participants had a localized disease status (60%). The mean self-perceived health status during the last week was 4.6 (with response options ranging from “1 = very poor” to “7 = excellent”) with a median of 5.0. About one-third of participants (32%) reported having no comorbidities. The most common reported comorbidities were heart diseases or circulation disturbances (13%), followed by endocrine disorders (8%).

**Table 1:** Sociodemographic and medical characteristics of the participants (*N* = 399)

	n	%
Age in Years		
Mean (SD)	394	57.1 (15.7)
Sex		
Female	170	42.6
Male	228	57.1
Missing values	1	0.3
Highest educational degree		
University	126	31.6
13 years of school education	75	18.8
10 years of school education	119	29.8
8 or 9 years of school education	72	18.0
No school certificate after 8 or 9 years of school education	3	0.8
Missing values	4	1.0
Marital Status		
Married, committed relationship	257	64.4
Single	79	19.8
Widowed	24	6.0
Divorced/living separated	36	9.2
Missing values	3	0.8
Professional situation		
Retired	173	43.3
Working full-time	127	31.8
Working part-time	37	9.4
Unemployed	20	5.0
Other	40	10.0
Missing values	2	0.5
Treatment setting		
Outpatient	332	83.2
Inpatient	64	16.0
Missing values	3	0.8
Cancer type ^a^		
Lymphoma	59	14.8
Breast	53	13.3
Prostate	50	12.5
Leukemia	37	9.3
Lung	28	7.0
Brain	25	6.3
Myeloma	19	4.8
Mouth, throat, esophagus	18	4.5
Colon	18	4.5
Pancreas	18	4.5
Other	174	43.9
Missing values	15	3.8
Self-perceived Health Status ^b^		
Mean (SD)	394	4.6 (1.3)
Comorbidities ^a^		
No comorbidity	127	32.2
Heart diseases or diseases of the circulatory system	52	13.2
Endocrine diseases	33	8.4
Diseases of the musculoskeletal system and inflammatory diseases	31	7.8
Diseases of the respiratory system	31	7.8
Skin diseases	23	5.8
Other diseases	98	24.8

^a^multiple selection possible

^b^range from 1 = “very poor” to 7 = “excellent”

### Item analysis

Table 2 shows the results of the items analysis. Sixteen items had less than 5% missing values. Additionally, item 16 had the most missing values, with 35 participants not completing it. Item 2 had zero missing values. The means of all 18 items ranged from 3.50 (item 16) to 4.59 (items 2). The corrected item-total correlation ranged from 0.36 to 0.77. Item 16 and 18 had a corrected item-total correlation below 0.40 [32]. All 18 items had a negative skew, indicating that most of the scores are above the mean [31]. None of the skew scores were above minus three [31]. Item 2 and item 17 had high skewness, with 66% or 51% providing a ‘Strongly Agree’ response, respectively. The other items did not show any ceiling effects.

**Table 2:** Psychometric properties for all 18 questions in the PSCC-G (N = 399)

Item	Acceptance (% missing)	Mean	SD	Corrected item-total correlations	Skewness	Ceiling effect (%)
1. I felt that my health concerns were understood	0.8	4.30	0.81	.68	−1.24	46.9
2. I felt that I was treated with courtesy and respect	0.0	4.59	0.67	.56	−1.97	66.4
3. I felt included in decisions about my health	1.5	4.12	0.99	.62	−1.15	42.6
4. I was told how to take care of myself	1.8	3.71	1.19	.66	−0.65	30.6
5. I felt encouraged to talk about my personal health concerns	1.8	3.85	1.14	.66	−0.86	34.3
6. I felt I had enough time with my doctor	1.8	4.03	1.07	.70	−1.18	39.1
7. My questions were answered to my satisfaction	0.8	4.17	0.96	.77	−1.26	44.1
8. Making an appointment was easy	1.0	4.16	0.96	.45	−1.13	45.1
9. I knew what the next step in my care would be	2.3	4.24	0.92	.60	−1.29	47.9
10. I feel confident in how I deal with the health care system	1.5	3.85	1.01	.58	−0.80	27.8
11. I was able to get the advice I needed about my health issue	1.5	4.09	0.87	.77	−0.93	35.6
12. I knew who to contact when I had a question	1.3	4.14	0.98	.65	−1.17	43.6
13. I received all service I needed	4.2	4.36	0.82	.61	−1.59	49.9
14. I am satisfied with the care I received	2.0	4.34	0.80	.71	−1.34	48.9
15. The doctors seemed to communicate well about my care	1.5	4.17	1.01	.74	−1.19	47.8
16. I received high quality care from my regular doctor	8.7	3.50	1.42	.36	−0.57	29.8
17. I received high quality care from my specialist	4.5	4.31	0.94	.62	−1.65	50.6
18. My regular doctor was informed about the results of the test I got	5.0	4.06	1.20	.36	−1.31	45.9

### Confirmative factor analysis

CFA was conducted on the 18 items of the PSCC-G with all items loading onto a single latent variable. Fit indices values were found to be: *χ*^2^ (df = 135) = 651.06, *p* < .001, CFI = .86, TLI = .84, RMSEA = .10 with 90% CI = .09–.11, SRMR = .06. Fifteen items had good, very good or excellent loadings (standardized regression weights) on the one factor. Item 16 and item 18 had poor loadings and item 8 had a fair loading [34].

### Explorative factor analysis

The Kaiser-Meyer-Olkin (KMO) measure of sampling adequacy, KMO = .93, indicated that the 18 items had an adequate common variance for factor analysis. The results of the initial principal axis analysis revealed three factors with eigenvalues above 1.0 (8.27, 1.29, 1.03), which explained 43.40, 4.34, and 3.13% of the total cumulative variance (50.87%). The scree-plot showed a steep cut-off from the primary factor to the secondary factor, which suggested the extraction of one primary factor. See Table 3 for the individual loadings for the one-factor solution. Briefly, 15 items had good, very good, or excellent factor loadings. Additionally, the extraction factor showed that Item 8 had a fair factor loading, and Item 16 and Item 18 had poor factor loadings [37].

**Table 3:** Factor loadings of a principal axis EFA (one factor solution)

No	Item	Factor 1
7.	My questions were answered to my satisfaction	.81
11.	I was able to get the advice I needed about my health issue	.80
15.	The doctors seemed to communicate well about my care	.77
14.	I am satisfied with the care I received	.76
6.	I felt I had enough time with my doctor	.75
1.	I felt that my health concerns were understood	.72
12.	I knew who to contact when I had a question	.68
4.	I was told how to take care of myself	.68
5.	I felt encouraged to talk about my personal health concerns	.68
3.	I felt included in decisions about my health	.66
13.	I received all the service I needed	.64
9.	I knew what the next step in my care would be	.63
17.	I received high quality care from my specialist	.62
10.	I feel confident in how I deal with the health care system	.61
2.	I felt that I was treated with courtesy and respect	.60
8.	Making an appointment was easy	.47
16.	I received high quality care from my regular doctor	.35
18.	My regular doctor was informed about the results of the test I got	.35

We further tested the PSCC-G as a one-dimensional scale based on the results of the scree-test of the EFA and the results of the CFA.

### Internal consistency and convergent validity of the PSCC-G

Our analysis revealed a Cronbach alpha of 0.92 for the PSCC-G. The score on the PSCC-G correlated significantly (*p* < .01) with the Satisfaction with the information provided by physicians subscale of RESPERES-G questionnaire (Pearson’s r = .57), and the co-management and shared decision making (Pearson’s r = .79), nursing staff and other practice assistants (Pearson’s r = .54), and involvement of family members and friends (Pearson’s r = .58) subscales of the PASQOC.

### Regression analysis predicting PSCC-G scores

The findings showed that the model explained approximately 17% of the variance in PS (Table 4). Sex, age, and perceived health status significantly predicted the PSCC-G total score. Specifically, being male, older, and having a higher self-perceived health status were associated with higher scores on the PSCC-G, indicating higher satisfaction with cancer care.

**Table 4:** Results of Multiple Regression Analysis for Variables predicting Patients’ Satisfaction (*N* = 379)

	B	SE	Exp (B)	p
Female sex	−2.50	1.10	−0.10	.02
Age	0.23	0.04	0.31	<.001
Living with partner (vs living alone)	1.21	1.18	0.05	.30
Perceived health status	2.40	0.41	0.27	<.001
University degree (vs. 9 years of school education)	−0.58	1.62	−0.02	.72
13 years of school education (vs. 9 years)	.83	1.83	0.03	.65
10 years of school education (vs 9 years)	1.663	1.62	0.07	.30
Inpatient treatment (vs outpatient treatment)	−2.11	1.47	−.07	.15

Adjusted R^2^ = .17 (*p* < .001)

## Discussion

Overall, the results indicate that PSCC-G is a *psychometrically valid* one-dimensional instrument, which is sufficiently able to measure cancer patients’ satisfaction with cancer-related care in inpatient and outpatient settings.

The translation of the questionnaire had followed the comprehensive EORTC guidelines. As a result, only small adjustments were necessary, indicating that the PSCC is already a questionnaire specific for cancer care, which appears to be transferable to different languages and cultures without larger difficulty.

The PSCC-G was *well accepted* in the study sample, which indicates an appropriate understandable translation of the questionnaire. Items 16 and 18 were the only items with more than 5% missing values [30].

The results revealed a sufficient *convergent validity* of the PSCC-G. The correlation with conceptually related constructs were all above r = .50 [38]. All items had good *corrected item-total correlations* except for items 16 and 18 [32]. Consistent with previous satisfaction measures, we observed a skewing or tendency towards the higher end of satisfaction [20, 26, 39]. Items 2 and 17 showed a pronounced *ceiling effect*. However, the skewness values of the two items do not meet the criteria to classify them as extreme [31]. Possible strategies for the reduction of the ceiling effect when measuring PS are among others the utilization of visual analogue scales [40] or changing the response format to a format with e.g. 3 positive and 2 negative choices [41]. The *predicted one-factor structure* of the original English version [22] could not be fully confirmed by the CFA. The fit indices for CFA using a one-dimensional model were close to but did not meet suggested cut off values [33]. Fifteen items had good, very good, or excellent factor loadings, however items 16 and 18 had only poor loadings on the extracted factor of the EFA, which suggests that these items may form a further subscale [34, 37]. However, we still believe that the one-dimensional model is the best fit for the instrument for several reasons. The scree-plot suggested one primary factor. It is also not recommended to build a subscale with just two items [42, 43], the second factor only explained 4% of the variance and the one-dimensional instrument had a high internal consistency [44].

An explanation for the reoccurring difficulties with items 16 and 18, which examine the cooperation between health care professionals working in oncology and the patients’ regular doctors, might be related to the recruited patient population. About one-third of the participants were recruited in outpatient treatment while receiving their first-line therapy. These participants might not have been able to estimate the degree of cooperation among health care professionals as they have had no appointment with their general practitioner after being transferred rather recently to specific oncological care.

Our results suggest, that the PSCC-G can be utilized to measure satisfaction with cancer-related care in inpatient and outpatient facilities in all phases of the course of the disease and treatment, from screening to the completion of treatment. Given the lack of clear indications when the best moment for data collection is, we recommend that practical considerations should be taken into account to be able to include as many patients as possible. A potential field of application for the PSCC-G in research are intervention studies aiming at improving the quality of care for cancer patients with different types of cancer at different stages of their treatment. Additionally, the PSCC-G can be applied in cancer in- and outpatient care to inform the clinics about potential areas of improvement. For example, low scores on item 3 (“I felt included in decisions about my health”) could indicate that the treating physicians do not give enough importance to shared decision making to meet the needs of the patient. However, the interpretation of results of PS questionnaires in this context should take into account, that variance in PS is also determined by different demands, expectations, and values of the patients [2, 45]. Furthermore, it could be reasonable to additionally use patient-reported experience measures (PREMS) which are also recommended as an important indicator of the quality of care [2]. PREMS aim to collect information to what extent specific aspects of healthcare were provided and are thus more independent of the patient’s perspective [46, 47].

Compared to generic questionnaires the PSCC-G as a disease-specific questionnaire is more likely to be sensitive to detect small but important differences between cancer patients’ satisfaction with cancer-related care [48–50]. Compared to the few existing and quite extensive PS questionnaires available in Germany it allows to compare cancer patients’ satisfaction regardless of cancer type [17, 26], stage [18, 19], and type of treatment and treatment setting [9, 20, 21, 27]. Broader eligibility criteria were used in our study than in the English [22] and Spanish validation studies [23], which led to a more heterogeneous patient sample.

One main strength of this validation study is the large and heterogeneous study sample. By conducting a power analysis before data collection, we were able to recruit enough patients to reach sufficient statistical power for all planned and conducted analyses. Additionally, we think that heterogeneity may lead to higher generalizability of the results.

However, several limitations have to be discussed. First, the response scale of two instruments were changed because patients complained about the format during the pilot testing or had difficulty filling out the questionnaire. In the case of the PSCC-G, we changed the direction of the response scale from a descending (‘strongly agree’ to ‘strongly disagree’) to an ascending (strongly disagree to strongly agree) direction. Using an ascending direction might lead to slightly lower satisfaction scores [51], possibly because of pseudoneglect, which is an attentional bias in participants that makes the left-sided features of a stimulus more salient than those on the right [52]. However, we think that potentially lower satisfaction scores only have a small influence on the validity of the PSCC-G. Lower satisfaction scores would have changed the results of the means, diminish skewness and ceiling effects, but would have had very little impact on the results of the factor analysis or convergent validity. In the case of the PASQOC we changed the answer scales due to recommendations by the patients of the pilot study to a consistent five-point response scaling. This might have had an impact on the answers and thus on the validity of the subscales of the PASQOC. Additional analyses showed that at least the internal consistency of all three subscales is still very high (co-management and shared decision making (6 items): α = .91; nursing staff and other practice assistants (3 items): α = .84; involvement of family members and friends (3 items): α = .84).

*Second*, because the response rate was not recorded, there might be a response bias towards patients, who are willing and capable to participate in a validation study, where it is necessary to answer a comprehensive set of questionnaires. This might have led to a sample with more patients with a higher education, thus potentially limiting the generalizability of the results, as patients with a high education might be more likely to report higher satisfaction with cancer care [7]. Nonetheless, the results of the multiple regression analysis did not indicate education to be a factor associated with the participants’ satisfaction. *Third*, after consultation with the medical staff on the wards and outpatient clinics, patients in poor medical condition were mostly not included in the study. Since cancer patients with a lower health status might report lower satisfaction with care [7, 11], the satisfaction scores of the PSCC-G in this study were probably lower than the satisfaction scores of the entire population of cancer patients in the included clinics and wards.

*Fourth*, the time since the cancer diagnosis of the participants has not been recorded. As the PSCC-G, PSCC, and PSCC-SP were designed to address the broad domain of cancer-related care ranging from diagnostic testing to treatment process and completion [22, 23], it could be interesting to longitudinally compare satisfaction throughout the treatment.

*Fifth*, the translation of a questionnaire is always a potential source of bias. The main issue with translating the PSCC into German was the word “care”. It is a generic term with multiple meanings and no exact equivalent in the German language. To minimize a potential translation bias we strictly adhered to the guidelines of the European Organization for Research and Treatment of Cancer (EORTC) and we pilot-tested the PSCC-G to identify potential misunderstandings [25]. In the pilot study, participants were asked to think aloud while completing the PSCC and we were under the impression that the term “care” was interpreted in the way it was intended.

## Conclusion

All things considered; the PSCC-G may prove to be useful for future investigations of PS in many German cancer patients. Future studies should examine the routine use of the PSCC-G in a larger clinical setting as well as its longitudinal use in different settings.

## Supplementary Information

**Additional file 1: Appendix 1.** Overview of all selected instruments and subscales.

## Data Availability

All of the data necessary for a meta-analysis are contained within the manuscript and its supplementary files. The datasets used and/or analysed during the current study are available from the corresponding author on reasonable request. The authors have full control of primary data and agree to allow the journal to review data if requested.

## Abbrevations

PSCC: Patient Satisfaction with Cancer Care
PSCC-G: Patient Satisfaction with Cancer Care-German
PASQOC: Patient Satisfaction and Quality in Oncology Care
RESPERES-60: Recherche Evaluative sur la Performance de Réseau de Sante
PS: Patient Satisfaction
EORTC: European Organization for Research and Treatment of Cancer
UKE: University Medical Center Hamburg-Eppendorf
UCCH: University Cancer Center Hamburg
PCC-SP: Patient Satisfaction with Cancer Care-Spanish
IBM: International Business Machines Corporation
SPSS: Statistical Package for the Social Sciences
SD: Standard Deviation
CFA: Confirmatory factor analysis
EFA: Exploratory factor analysis
CFI: Comparative-fit-index
RMSEA: Root mean square error of approximation
TLI: Tucker–Lewis Index
SRMR: Standardized Root Mean Square Residual
KMO: Kaiser-Meyer-Olkin
