# Supplementary material for: Psychometric evaluation of the German version of the Patient Satisfaction with Cancer-related Care questionnaire
Source: BMC Health Serv Res. 2020 Oct 27;20:983. doi: 10.1186/s12913-020-05838-7 (PMC7590742; doi:10.1186/s12913-020-05838-7)
Supplement: Supplementary file 1 — Additional file 1: Appendix 1. Overview of all selected instruments and subscales. [file 12913_2020_5838_MOESM1_ESM.docx]

| **No** | **selected Instruments** | **selected Subscales** |
| --- | --- | --- |
| 1. | German Translation of the PSCC | All 18 items |
| 2. | Recherché Evaluative sur la Performance de Réseau de Santé- German (RESPERES-G) | (1) Satisfaction with information provided by physicians |
| 3. | Patient Satisfaction and Quality in Oncological Care (PASCQOC) | (1) co-management and shared decision making  (2) nursing staff and other practice assistants  (3) Involvement of family members and friends. |
| 4. | EORTC QLQ-C30 | Overall health status item |

Appendix 1. Overview of all selected instruments and subscales
